# Genome Sequence of a Plant Growth-Promoting Rhizobacterium, Pseudomonas sp. Strain 31-12

**DOI:** 10.1128/MRA.00947-18

**Published:** 2018-08-16

**Authors:** Russell K. Hynes, Tim J. Dumonceaux, Jakkrapong Kangsopa, Jennifer R. Town

**Affiliations:** a Agriculture and Agri-Food Canada, Saskatoon Research and Development Centre, Saskatoon, Saskatchewan, Canada; b Department of Plant Science and Agricultural Resources, Faculty of Agriculture, Khon Kaen University, Khon Kaen, Thailand; University of California, Riverside

## Abstract

We present here a draft genome sequence of Pseudomonas sp. strain 31-12, a plant growth-promoting rhizobacterium of several crop plants that was isolated from the rhizosphere of corn in southern Ontario, Canada.

## ANNOUNCEMENT

The rhizosphere contains plant growth-promoting rhizobacteria (PGPRs), a group of microorganisms that positively impact plant growth and yield and negatively influence disease (1–3). Pseudomonas sp. strain 31-12 was isolated from the rhizosphere of corn and has characteristic plant growth promotion when applied as an inoculate to crop plants such as canola and alfalfa (4, 5).

Pseudomonas sp. strain 31-12 was grown from a single colony in half-strength Trypticase soy broth (TSB; Difco, Becton, Dickinson) at 25°C for 48 h with rotary shaking at 150 rpm. Genomic DNA (gDNA) was purified using the Wizard gDNA extraction kit (Promega) with 1 ml of overnight half-strength TSB culture as starting material. DNA was diluted to 2.5 ng/µl in 10 mM Tris-Cl buffer (pH 8.0) and then sheared to an average fragment size of ∼320 bp using sonication under high power with 30 cycles of 30 s on/30 s off with cooling to 4°C (Bioruptor 300, Diagenode). Genomic DNA was prepared for sequencing using the NEBNext Ultra DNA library prep kit for Illumina (New England Biolabs) and then sequenced on the Illumina MiSeq platform (600 cycles), generating 1.2 million paired reads. To facilitate a high-quality genome assembly, a second sequencing run was undertaken using the Oxford Nanopore Technologies (ONT) sequencing platform. Genomic DNA was purified using the Wizard gDNA extraction kit, and long fragments were size selected using AMPure XP beads (Beckman Coulter) at 0.45 (vol/vol), prepared for sequencing with a native 1D barcoding kit (ONT), and sequenced on a nanopore flow cell. ONT sequencing generated 22,981 reads with an average length of 9,819 bp (range, 219 to 139,994 bp) and quality score of 11.28. Illumina and ONT reads were coassembled using Unicycler version 0.4.4 with default parameters, yielding a finished (6) genome sequence with a single scaffold (average 34× coverage) and no gaps. No evidence was found of any plasmids in the sequencing data. The genome sequence was annotated using the NCBI Prokaryotic Genome Annotation Pipeline version 3.1 (7).

The assembled genome of Pseudomonas sp. strain 31-12 consisted of 6,730,253 bp and had a GC content of 59.1%. Moreover, 6,011 protein-coding genes were observed in the genome, as well as 7 genes encoding 5S rRNA, 6 identical genes encoding 16S rRNA, 6 genes encoding 23S rRNA, and 67 genes encoding tRNA. An additional 280 pseudogenes were annotated in the genomic sequence.

The 16S rRNA-encoding sequence of Pseudomonas sp. strain 31-12 was identical to those of several previously described Pseudomonas strains and species. Phylogenetic analysis of an alternative phylogenetic marker, *cpn60* (8), revealed that Pseudomonas sp. strain 31-12 clustered with, but was distinct from, P. mandelii 36MFCvi1.1 (GenBank accession number KB906330) and P. umsongensis 20MFCvi1.1 (KB898552). Examination of the whole-genome nucleotide sequence using JSpeciesWS (9) revealed that Pseudomonas sp. strain 31-12 was below the specified sequence identity threshold for inclusion in any previously described species of Pseudomonas. This suggests the possibility that the plant growth-promoting bacterium Pseudomonas sp. strain 31-12 may be a novel species within the genus Pseudomonas.

### Data availability.

The data for this complete genome sequence have been deposited at DDBJ/EMBL/GenBank under the accession number CP029482. Raw sequence reads (ONT and Illumina) have been deposited to the NCBI Sequence Read Archive under the accession number SRP153420.
